# Retraction: ARHGAP24 inhibits cell cycle progression, induces apoptosis and suppresses invasion in renal cell carcinoma

**DOI:** 10.18632/oncotarget.28749

**Published:** 2025-06-17

**Authors:** Gaosi Xu, Xiongbing Lu, Tianlun Huang, Jie Fan

**Affiliations:** ^1^Department of Nephrology, The Second Affiliated Hospital of Nanchang University, Nanchang, China; ^2^Department of Urology, The Second Affiliated Hospital of Nanchang University, Nanchang, China; ^*^These authors have contributed equally to this work

**This article has been retracted:** This decision follows an investigation by Oncotarget into concerns raised by a third party. Our Image Forensics analysis found that the GAPDH Western blot image in Figure 2D was previously used as a loading control in Figure 2C in an unrelated paper [1]. The Western blot for CDK2 in Figure 3F was earlier published as an MMP 2 blot in Figure 4C [2] and as a b-catenin blot in Figure 4C [3] of unrelated papers that have already been retracted [2, 3]. In addition, Figure 5B contains an invasion image of Caki-2 cells (renal cell carcinoma) that was also found in an earlier published paper [4] in Figure 6C, where it presented transwell assay data for ECA-109 human esophageal carcinoma cells. Furthermore, several images from this paper have been subsequently used in publications by different research groups in other journals. The corresponding author, Gaosi Xu, has acknowledged these duplications and requested a retraction. While multiple attempts were made to contact the other authors for their acknowledgment of this retraction, no responses were received. Consequently, based on these findings and the corresponding author’s request, the Editorial decision has been made to retract this paper.

Original article: Oncotarget. 2016; 7:51829–51839. 51829-51839. https://doi.org/10.18632/oncotarget.10386
